# Decline in COVID-19 Hospitalization Growth Rates Associated with Statewide Mask Mandates — 10 States, March–October 2020

**DOI:** 10.15585/mmwr.mm7006e2

**Published:** 2021-02-12

**Authors:** Heesoo Joo, Gabrielle F. Miller, Gregory Sunshine, Maxim Gakh, Jamison Pike, Fiona P. Havers, Lindsay Kim, Regen Weber, Sebnem Dugmeoglu, Christina Watson, Fátima Coronado

**Affiliations:** ^1^CDC COVID-19 Response Team; ^2^University of Nevada, Las Vegas.

SARS-CoV-2, the virus that causes coronavirus disease 2019 (COVID-19), is transmitted predominantly by respiratory droplets generated when infected persons cough, sneeze, spit, sing, talk, or breathe. CDC recommends community use of face masks to prevent transmission of SARS-CoV-2 (*1*). As of October 22, 2020, statewide mask mandates were in effect in 33 states and the District of Columbia (*2*). This study examined whether implementation of statewide mask mandates was associated with COVID-19–associated hospitalization growth rates among different age groups in 10 sites participating in the COVID-19–Associated Hospitalization Surveillance Network (COVID-NET) in states that issued statewide mask mandates during March 1–October 17, 2020. Regression analysis demonstrated that weekly hospitalization growth rates declined by 2.9 percentage points (95% confidence interval [CI] = 0.3–5.5) among adults aged 40–64 years during the first 2 weeks after implementing statewide mask mandates. After mask mandates had been implemented for ≥3 weeks, hospitalization growth rates declined by 5.5 percentage points among persons aged 18–39 years (95% CI = 0.6–10.4) and those aged 40–64 years (95% CI = 0.8–10.2). Statewide mask mandates might be associated with reductions in SARS-CoV-2 transmission and might contribute to reductions in COVID-19 hospitalization growth rates, compared with growth rates during <4 weeks before implementation of the mandate and the implementation week. Mask-wearing is a component of a multipronged strategy to decrease exposure to and transmission of SARS-CoV-2 and reduce strain on the health care system, with likely direct effects on COVID-19 morbidity and associated mortality.

Data on statewide mask mandates during March 1–October 22, 2020, were obtained by CDC and the University of Nevada, Las Vegas, from state government websites containing executive or administrative orders, which were analyzed and coded to extract effective dates of statewide mask mandates. A statewide mask mandate was defined as the requirement that persons operating in a personal capacity (i.e., not limited to specific professions or employees) wear a mask 1) anywhere outside their home or 2) in retail businesses and in restaurants or food establishments. All coding and analyses underwent secondary review and quality assurance checks by two or more raters; upon agreement among all raters, coding and analyses were published in a freely available data set (*2*).

Cumulative COVID-19–associated hospitalization rates for each week during March 1–October 17, 2020, (33 weeks) were obtained from COVID-NET, a population-based surveillance system (*3*). COVID-NET provides laboratory-confirmed, COVID-19–associated hospitalization rates (hospitalizations per 100,000 persons) in 99 counties located in 14 states, commencing the week of March 1, 2020* (*4*). Certain counties in each state participate in COVID-NET, except Maryland, where all counties participate. A group of counties participating in COVID-NET within a state is termed a site. Sites in states that did not have statewide mask mandates during March 1–October 17, 2020, were excluded from the analyses. For analyses, cumulative hospitalization rates for each week of the study period for seven age cohorts (adults aged 18–29, 30–39, 40–49, 50–64, 65–74, 75–84, and ≥85 years) were aggregated into three age groups (18–39, 40–64, and ≥65 years)^†^; sites with a cumulative hospitalization rate of zero per 100,000 persons were imputed to 0.1 per 100,000. Hospitalizations among children and adolescents aged <18 years were not included because few hospitalizations were reported among this age group during the study period.

The outcome was the hospitalization growth rate, defined as the weekly percentage change in cumulative COVID-19 hospitalizations per 100,000 persons. The weekly percentage change was calculated as the difference of logarithms in cumulative COVID-19 hospitalization rates by week.^§^ The association between mask mandates and COVID-19–associated hospitalization growth rates was measured using a time-based categorical variable with four mutually exclusive categories based on the week (Sunday through Saturday), with the effective date of the mask mandate (“implementation week”) characterized as follows: ≥4 weeks before the implementation week; <4 weeks before the implementation week (reference); <3 weeks after the implementation week; and ≥3 weeks after the implementation week.^¶^ Week zero (implementation week) was defined as the week that included the date the mask mandate went into effect and was included in the reference period. The hospitalization rate ≥4 weeks before implementation of the mask mandate was compared with that during the reference period to test whether sites with mask mandates had differential trends in COVID-19–associated hospitalization rates before issuance of mask mandates

This study used a regression model with panel data to compare COVID-19–associated hospitalization growth rates at COVID-NET sites with mandates before and after the dates that statewide mask mandates became effective (*5*). Using hospitalization growth rates before mask mandates were implemented (i.e., the reference period: <4 weeks before the implementation week and the implementation week), the model predicted hospitalization growth rates after mask mandates, assuming mandates had not been implemented. Then the model compared the predicted values with the observed hospitalization growth rates after mask mandates were implemented. The study controlled for mask mandates, state, age group, and time (i.e., week of the year).** The study also controlled for statewide closing and reopening as determined by the date of stay-at-home orders and business closures (Supplementary Table, https://stacks.cdc.gov/view/cdc/101127).^††^ P-values <0.05 were considered statistically significant. Analyses were conducted separately for three age groups (18–39, 40–64, and ≥65 years) and for all adults aged ≥18 years using Stata software (version 16.1; StataCorp). This study was reviewed by CDC and was conducted consistent with applicable federal law and CDC policy.^§§^

Ten of the 14 COVID-NET participating sites were in states that had issued statewide mask mandates since March 2020 (Table 1). The overall COVID-19–associated hospitalization growth rates among all adults declined 2.4 percentage points (p-value = 0.04) <3 weeks after the implementation week and declined 4.9 percentage points (p-value <0.01) during the period ≥3 weeks after the implementation week (Table 2). The declines were statistically significant.

**TABLE 1:** Effective dates of statewide mask mandates — 10 COVID-19–Associated Hospitalization Surveillance Network sites with statewide mask mandates, March–October 2020

State	Effective date of statewide mask mandate	Source
California	Jun 18, 2020	California Health Order (Jun 18, 2020) https://www.countyofnapa.org/DocumentCenter/View/17945/Guidance-for-Face-Coverings_06-18-2020)
Colorado	Jul 16, 2020	Colorado Executive Order No. D 2020–138 (Jul 16, 2020) https://www.colorado.gov/governor/sites/default/files/inline-files/D%202020%20138%20Mask%20Order.pdf)
Connecticut	Apr 20, 2020	Connecticut Executive Order No. 7BB (Apr 17, 2020) (https://portal.ct.gov/-/media/Office-of-the-Governor/Executive-Orders/Lamont-Executive-Orders/Executive-Order-No-7BB.pdf)
Maryland	Apr 18, 2020	Maryland Executive Order No. 20–04–15–01 (Apr 15, 2020) (https://governor.maryland.gov/wp-content/uploads/2020/04/Masks-and-Physical-Distancing-4.15.20.pdf)
Michigan*	Apr 26, 2020	Michigan Executive Order No. 2020–59 (Apr 24, 2020) (https://content.govdelivery.com/attachments/MIEOG/2020/04/24/file_attachments/1435194/EO%202020-59.pdf)
Minnesota	Jul 24, 2020	Minnesota Emergency Executive Order 20–81 (Jul 22, 2020) (https://mn.gov/governor/assets/EO%2020-81%20Final%20Filed_tcm1055-441323.pdf)
New Mexico	Jun 1, 2020	New Mexico Health Order (Jun 1, 2020) (https://cv.nmhealth.org/wp-content/uploads/2020/06/060120-PHO.pdf)
New York	Apr 17, 2020	New York Executive Order No. 202.17 (Apr 15, 2020) (https://www.governor.ny.gov/news/no-20217-continuing-temporary-suspension-and-modification-laws-relating-disaster-emergency)
Ohio	Jul 23, 2020	Ohio Health Order (Jul 23, 2020) (https://coronavirus.ohio.gov/static/publicorders/Directors-Order-Facial-Coverings-throughout-State-Ohio.pdf)
Oregon	Jul 1, 2020	Oregon Health Order (Jun 30, 2020) (https://web.archive.org/web/20200702101516/https://sharedsystems.dhsoha.state.or.us/DHSForms/Served/le2288K.pdf)

**Abbreviation:** COVID-19 = coronavirus disease 2019.

* Because of a ruling from Michigan’s supreme court, a 3-day lapse in Michigan’s statewide mask mandate occurred during October 2–4. The analyses did not consider this lapse. All other statewide mask mandates were continuous throughout the study period.

**TABLE 2:** Estimated association between mask mandates and COVID-19–associated hospitalization growth rates in sites with statewide mask mandates, by age group — 10 COVID-19–Associated Hospitalization Surveillance Network sites,*^,^^†^ March–October 2020

Time relative to week mask mandate was implemented	All (≥18 yrs)		18–39 yrs		40–64 yrs		≥65 yrs	
	Percentage point change* (95% CI)	p-value	Percentage point change* (95% CI)	p-value	Percentage point change* (95% CI)	p-value	Percentage point change* (95% CI)	p-value
≥4 weeks before	−4.3 (−10.5 to 1.9)	0.17	−4.7 (−16.9 to 7.5)	0.43	−4.0 (−13.3 to 5.3)	0.38	−5.3 (−14.9 to 4.3)	0.27
<4 weeks before^§^	Referent	—	Referent	—	Referent	—	Referent	—
<3 weeks after	−2.4 (−4.7 to −0.1)	0.04	−2.1 (−6.4 to 2.2)	0.31	−2.9 (−5.5 to −0.3)	0.03	−1.1 (−3.9 to 1.6)	0.41
≥3 weeks after	−4.9 (−8.5 to −1.2)	<0.01	−5.5 (−10.4 to −0.6)	0.03	−5.5 (−10.2 to −0.8)	0.02	−0.5 (−5.2 to 4.1)	0.83

**Abbreviations:** CI = confidence interval; COVID-19 = coronavirus disease 2019.

* Percentage points are coefficients from the regression models. Reported numbers are from regression models, which controlled for state, age group, time (week), and statewide closing and reopening.

^†^ California, Colorado, Connecticut, Maryland, Michigan, Minnesota, New Mexico, New York, Ohio, and Oregon.

^§^ This period includes the implementation week (i.e., week zero).

Among persons aged 18–39 years, the hospitalization growth rates <3 weeks after the implementation week were lower than were those during the <4 weeks before the implementation week and the implementation week (reference period) when no mask mandate existed, but the estimated percentage point difference (–2.1) was not statistically significant (p-value = 0.31) (Figure) (Table 2). However, in this population, mask mandates were associated with a statistically significant 5.5 percentage-point decline in COVID-19 hospitalization growth rates (p-value = 0.03) ≥3 weeks after the implementation week. Among adults aged 40–64 years, mask mandates were associated with a 2.9 percentage-point reduction in COVID-19 hospitalization growth rates (p-value = 0.03) <3 weeks after the implementation week. Hospitalization growth rates declined by 5.5 percentage points (p-value = 0.02) during ≥3 weeks after the implementation week. Among adults aged ≥65 years, COVID-19 hospitalization growth rates declined <3 weeks after the implementation week (1.1 percentage points) and ≥3 weeks after the implementation week (0.5 percentage points); however, the declines were not statistically significant.

**FIGURE:**
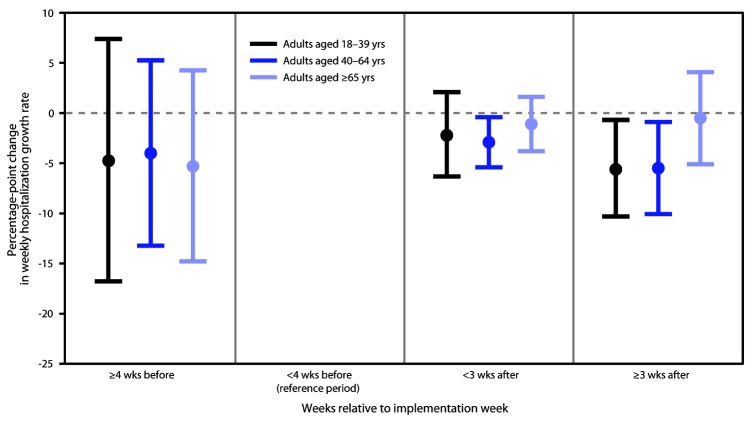
Estimates of association between implementation of statewide mask mandates and laboratory-confirmed COVID-19–associated hospitalization growth rates,*^,†,§^ by age group — 10 COVID-19–Associated Hospitalization Surveillance Network sites^¶^ with statewide mask mandates, March–October 2020 **Abbreviation**: COVID-19 = coronavirus disease 2019. * With error bars indicating 95% confidence intervals. ^†^ Relative to <4 weeks before implementation week (reference period, which includes the implementation week). ^§^ Reported numbers are coefficients from the regression models, which controlled state, age group, time (week), and statewide closing and reopening. ^¶^ California, Colorado, Connecticut, Maryland, Michigan, Minnesota, New Mexico, New York, Ohio, and Oregon.

In the ≥4 weeks before the implementation week, COVID-19–associated hospitalization growth rates were lower than were those <4 weeks before the implementation week and during the implementation week (reference). However, the percentage point differences were not statistically significant.

## Discussion

Masks are intended to reduce emission of virus-laden respiratory droplets, which is especially relevant for persons who are infected with SARS-CoV-2 but are asymptomatic or presymptomatic; masks also help reduce inhalation of respiratory droplets by the wearer (*1*). Findings from this study suggest that statewide mask mandates were associated with statistically significant declines in weekly COVID-19 hospitalization growth rates for adults aged 40–64 years <3 weeks after the week that the mandate was implemented, and for adults aged 18–64 years ≥3 weeks after the implementation week. The declines in hospitalization growth rates <3 weeks after the implementation week are consistent with the incubation period of SARS-CoV-2; in a report based on an analysis of publicly reported confirmed COVID-19 cases, the median estimated incubation period was 5.1 days, and most symptomatic patients reported symptoms within 11.5 days after exposure (*6*). Therefore, <3 weeks after the implementation of mask mandate would be long enough to identify an association between mask mandates and COVID-19–associated hospitalization growth rates. Previous studies have shown that the various physical distancing measures, including mask mandates, were associated with immediate declines in COVID-19 case growth rates (*5*,*7*).

This study did not demonstrate a statistically significant decline in COVID-19–associated hospitalization growth rates for adults aged ≥65 years, suggesting that there might have been less of a decline in this age group, compared with that of other adults, although CIs were wide. A study conducted during May 2020 indicated that approximately 70% of U.S. adults aged ≥65 years reported always wearing a mask in public, compared with only 44% of those aged 18–24 years (*8*). As a result, statewide mask mandates might have had a lesser impact on the masking behaviors of adults aged ≥65 years, compared with behaviors among other adults because of relatively high baseline level of mask use among this age group during the reference period (i.e., <4 weeks before the implementation week and the implementation week).

Declines in hospitalization growth rates during March 1–October 17, 2020, might also have resulted in a substantial decrease in health care costs associated with COVID-19. CDC has determined that COVID-19–related hospital costs per adult hospitalization varied from $8,400 in a general ward to >$50,000 in an intensive care unit with a ventilator (*9*). Because COVID-19 can lead to prolonged illness and require long-term treatment (*10*), the expected savings associated with the decline in hospitalization rates could be much higher than these reduced hospital costs associated with COVID-19.

The findings in this report are subject to at least four limitations. First, the model did not control for other policies that might affect hospitalization growth rates, including school closing and physical distancing recommendations; however, it did control for the dates of statewide closing and reopening, based on statewide stay-at-home orders and business closures. Second, these findings are limited to state-issued statewide mask mandates and do not account for local variability, such as county-level mask mandates.^¶¶^ Third, the findings are based on sites participating in COVID-NET and are limited to persons aged ≥18 years and therefore might not be generalizable to the entire U.S. population. Finally, it was assumed that the estimated effect in hospitalization growth rates after mask mandate implementation week did not depend on the issuance dates (e.g., Monday versus Friday), although number of days after the issuance of mask mandates in week zero varied by issuance date. Also, it was assumed that the mask mandates could not affect the hospitalization growth rates during the implementation week.

At the individual level, the prevention benefit of using a mask increases as more persons use masks consistently and correctly. Studies have confirmed the benefit of masking for SARS-CoV-2 control; each study demonstrated that, after implementation of directives from organizational or political leadership for universal masking, new infections decreased significantly (*1*). This study supports community masking to reduce the transmission of SARS-CoV-2. It also demonstrates that statewide mask mandates were associated with a reduction in COVID-19–associated hospitalization growth rates among adults aged 18–64 years and might affect age groups differently. Mask-wearing is part of a multipronged application of evidence-based strategies that prevent the transmission of SARS-CoV-2; wearing a mask reduces exposure, transmission, and strain on the health care system with likely direct effects on COVID-19 morbidity and associated mortality (*1*).

**Summary**

**What is already known about this topic?**

Wearing masks is recommended to mitigate the spread of COVID-19.

**What is added by this report?**

During March 22**–**October 17, 2020, 10 sites participating in the COVID-19–Associated Hospitalization Surveillance Network in states with statewide mask mandates reported a decline in weekly COVID-19–associated hospitalization growth rates by up to 5.5 percentage points for adults aged 18–64 years after mandate implementation, compared with growth rates during the 4 weeks preceding implementation of the mandate.

**What are the implications for public health practice?**

Mask-wearing is a component of a multipronged strategy to decrease exposure to and transmission of SARS-CoV-2 and reduce strain on the health care system, with likely direct effects on COVID-19 morbidity and associated mortality.

## Footnotes

* Counties by state in COVID-NET surveillance: California (Alameda, Contra Costa, and San Francisco counties); Colorado (Adams, Arapahoe, Denver, Douglas, and Jefferson counties); Connecticut (New Haven and Middlesex counties); Georgia (Clayton, Cobb, DeKalb, Douglas, Fulton, Gwinnett, Newton, and Rockdale counties); Iowa (one county represented); Maryland (Allegany, Anne Arundel, Baltimore, Baltimore City, Calvert, Caroline, Carroll, Cecil, Charles, Dorchester, Frederick, Garrett, Harford, Howard, Kent, Montgomery, Prince George’s, Queen Anne’s, St. Mary’s, Somerset, Talbot, Washington, Wicomico, and Worcester counties); Michigan (Clinton, Eaton, Genesee, Ingham, and Washtenaw counties); Minnesota (Anoka, Carver, Dakota, Hennepin, Ramsey, Scott, and Washington counties); New Mexico (Bernalillo, Chaves, Doña Ana, Grant, Luna, San Juan, and Santa Fe counties); New York (Albany, Columbia, Genesee, Greene, Livingston, Monroe, Montgomery, Ontario, Orleans, Rensselaer, Saratoga, Schenectady, Schoharie, Wayne, and Yates counties); Ohio (Delaware, Fairfield, Franklin, Hocking, Licking, Madison, Morrow, Perry, Pickaway, and Union counties); Oregon (Clackamas, Multnomah, and Washington counties); Tennessee (Cheatham, Davidson, Dickson, Robertson, Rutherford, Sumner, Williamson, and Wilson counties); and Utah (Salt Lake County).

† The analysis for adults aged 18–39 years used observations of adults aged 18–29 and 30–39 years; the analysis for adults aged 40–64 years used observations of adults aged 40–49 and 50–64 years; the analysis for adults aged ≥65 years used observations of adults aged 65–74, 75–84, and ≥85 years.

§ Weekly cumulative hospitalization growth rate (HGrowth*_ast_*) for age cohort *a* in site *s* during week *t* is defined as the weekly percentage change in COVID-19 hospitalizations per 100,000 persons, estimated by HGrowth_ast_ = ((log (*HR_ast_*)-log (*HR_as(t_*_-1)_))×100, where *HR_ast_* = cumulative hospitalization rate per 100,000 population for age cohort *a* in site *s* in week *t.* The log of the cumulative hospitalization growth rate is similar to the log of the cumulative cases per week, as the denominators are equivalent.

¶ Each period might include different numbers of weeks by site. For ≥4 weeks before the implementation week (i.e., –4 or before), the maximum number of weeks included was 17 (–20 through –4), and the minimum was 3 (–6 through –4). For the periods of <4 weeks before the implementation week (i.e., –3 through 0), all sites have 4 weeks. For <3 weeks after the implementation week (i.e., 1 through 2), all sites have 2 weeks. For ≥3 weeks after the implementation week (i.e., 3 or after), the maximum number of weeks included is 24 (3 through 26), and the minimum is 10 (3 through 12).

** The event study design was adopted from a previous study (https://www.healthaffairs.org/doi/10.1377/hlthaff.2020.00818) and modified for the current analyses. Regression models used National Center for Health Statistics vintage 2018 bridged-race population estimates (https://www.cdc.gov/nchs/nvss/bridged_race.htm) for each site as analytic weights. The model used was a weighted least squares regression which accounted for heteroskedasticity by estimating the standard errors using age cohort-state clusters.

†† The date of the statewide closing was the earlier of 1) the date persons were required to stay home or 2) the date that restaurants were required to cease on-premises dining and that nonessential retail businesses were ordered to close. The date of the statewide reopening was the earlier of 1) the date the stay-at-home order was lifted or 2) the date that restaurants were allowed to resume on-premises consumption and that nonessential retail businesses were permitted to reopen.

§§ 45 C.F.R. part 46, 21 C.F.R. part 56; 42 U.S.C. Sect. 241(d); 5 U.S.C. Sect. 552a; 44 U.S.C. Sect. 501 et seq.

¶¶ Some states issued orders that applied to certain counties, and others authorized counties to apply for and receive variances from mitigation measures if certain thresholds were met (e.g., COVID-19 percentage of positive test results below a specified level in that county). Cities and counties might have also issued local mask mandates.
