# *Erratum:* Vol. 70, No. 6

**DOI:** 10.15585/mmwr.mm7008a4

**Published:** 2021-02-26

The report “Decline in COVID-19 Hospitalization Growth Rates Associated with Statewide Mask Mandates — 10 States, March–October 2020” contained several errors.

On page 212, in the first paragraph, the fifth sentence should have read “After mask mandates had been implemented for ≥3 weeks, hospitalization growth rates declined by **5.6** percentage points among persons aged 18–39 years (95% CI = **0.9**–10.4) and those aged 40–64 years (95% CI = **1.0**–10.2).”

On page 213, in the first complete paragraph of the right-hand column, the second sentence should have read “The overall COVID-19–associated hospitalization growth rates among all adults declined 2.4 percentage points (p-value = 0.04) <3 weeks after the implementation week and declined **5.0** percentage points (p-value <0.01) during the period ≥3 weeks after the implementation week (Table 2).”

**TABLE 2:** Estimated association between mask mandates and COVID-19–associated hospitalization growth rates in sites with statewide mask mandates, by age group — 10 COVID-19–Associated Hospitalization Surveillance Network sites,*^,†^ March–October 2020

Time relative to week mask mandate was implemented	All (≥18 yrs)		18–39 yrs		40–64 yrs		≥65 yrs	
	Percentage point change* (95% CI)	p-value	Percentage point change* (95% CI)	p-value	Percentage point change* (95% CI)	p-value	Percentage point change* (95% CI)	p-value
≥4 weeks before	−4.3 (**−10.6** to 1.9)	0.17	**−4.8** (**−17.0** to 7.5)	0.43	−4.0 (−13.3 to 5.3)	0.38	−5.3 (**−15.0** to **4.4**)	0.27
<4 weeks before^§^	Referent	—	Referent	—	Referent	—	Referent	—
<3 weeks after	−2.4 (−4.7 to −0.1)	0.04	**−2.2** (−6.4 to **2.1**)	**0.30**	−2.9 (−5.5 to −0.3)	0.03	**−1.2** (−3.9 to **1.5**)	**0.38**
≥3 weeks after	**−5.0** **(−8.6** to **−1.4)**	<0.01	**−5.6** (−10.4 to **−0.9**)	**0.02**	**−5.6** (−10.2 to **−1.0**)	0.02	**−0.7** (**−5.3** to **3.9**)	**0.76**

**Abbreviations:** CI = confidence interval; COVID-19 = coronavirus disease 2019.

* Percentage points are coefficients from the regression models. Reported numbers are from regression models, which controlled for state, age group, time (week), and statewide closing and reopening.

^†^ California, Colorado, Connecticut, Maryland, Michigan, Minnesota, New Mexico, New York, Ohio, and Oregon.

^§^ This period includes the implementation week (i.e., week zero).

On pages 213–214, the second complete paragraph of the right-hand column should have read “Among persons aged 18–39 years, the hospitalization growth rates <3 weeks after the implementation week were lower than were those during the <4 weeks before the implementation week and the implementation week (reference period) when no mask mandate existed, but the estimated percentage point difference (**–2.2**) was not statistically significant (p-value = **0.30**) (Figure) (Table 2). However, in this population, mask mandates were associated with a statistically significant **5.6** percentage-point decline in COVID-19 hospitalization growth rates (p-value = **0.02**) ≥3 weeks after the implementation week. Among adults aged 40–64 years, mask mandates were associated with a 2.9 percentage-point reduction in COVID-19 hospitalization growth rates (p-value = 0.03) <3 weeks after the implementation week. Hospitalization growth rates declined by **5.6** percentage points (p-value = 0.02) during ≥3 weeks after the implementation week. Among adults aged ≥65 years, COVID-19 hospitalization growth rates declined <3 weeks after the implementation week (**1.2** percentage points) and ≥3 weeks after the implementation week (**0.7** percentage points); however, the declines were not statistically significant.”

On page 214, there were multiple errors in Table 2. The corrected table is as follows:

On page 215, the second paragraph of the Summary should have read “During March 22**–**October 17, 2020, 10 sites participating in the COVID-19–Associated Hospitalization Surveillance Network in states with statewide mask mandates reported a decline in weekly COVID-19–associated hospitalization growth rates by up to **5.6** percentage points for adults aged 18–64 years after mandate implementation, compared with growth rates during the 4 weeks preceding implementation of the mandate.”

The Supplementary Table (https://stacks.cdc.gov/view/cdc/101127) should have listed the date of statewide reopening for Michigan as **June 1, 2020**.

